# Filler Injection Between the Superficial and Deep Temporal Fascia and Diameter of Superficial Temporal Arteries for Temple Augmentation: A Systematic Review and Meta‐Analysis

**DOI:** 10.1111/jocd.70632

**Published:** 2025-12-29

**Authors:** Amir Hashemloo, Maryam Milanifard

**Affiliations:** ^1^ Department of Medicine Shahid Beheshti University of Medical Sciences Tehran Iran; ^2^ Trauma and Injury Research Center, Student Research committee Iran University of Medical Sciences Tehran Iran

**Keywords:** cannula, hyaluronic acid filler, superficial temporal, temple augmentation, temporal fascia

## Abstract

**Background and Aim:**

With age, temple depressions appear due to age‐related atrophy of fatty tissue, leading to a prominent cheekbone and lateral rim of the eye socket. Hyaluronic acid fillers have become very popular in the temple area due to their simple procedure and reliable results. The present study was conducted with the aim of determining the effectiveness of filler injection between the superficial and deep temporal fascia and the diameter of superficial temporal arteries for temple enlargement.

**Method:**

The international databases PubMed, EMBASE, and Web of Science were searched using keywords aligned with the study objective up to July 2025. All articles were reviewed by two blinded, independent authors. STATA/MP.v17 (College Station, Texas, USA) was used to perform the analyses.

**Result:**

Based on the findings of seven included studies, the efficacy of HA filler injection in the STF and DTF area showed an efficacy of 85% (ES: 85%, 95% CI: 81%–91%). According to subgroup meta‐analysis, higher efficacy was observed with volume injection of 1.5 mL on each side (ES: 94%, 95% CI: 77%–100%). Subgroup meta‐analysis showed that the 18‐gauge cannula had the highest effectiveness (ES: 94%, 95% CI: 77%–100%).

**Conclusion:**

For temple augmentation, hyaluronic acid filler injection between the superficial and deep temporal fasciae is a highly effective and safe procedure; it is also recommended to inject filler in small volumes and use 18‐gauge and 21‐gauge cannulas to target the supraperiosteal and supra‐deep temporal fascia layers.

## 1 Introduction

To enlarge the temple, injection of hyaluronic acid (HA) filler into the temporal region is a suitable and less invasive option [1]. Studies have shown that after injection of HA filler, the temporal region increases in volume and makes it smoother; when injecting fillers into the temporal region, anatomical layers, vascular distribution, and local changes must be considered [2, 3, 4].

The most common reported side effects of injectable fillers in the temporal region are vascular complications [5]. Skin necrosis is observed as a result of filler injection into blood vessels or vascular compression [6]. To reduce vascular complications, anatomical information such as vascular distribution, routes, and diameters is of great importance [7].

In the temporal region, the superficial temporal artery is one of the important blood vessels to consider [8]. Evidence suggests that the anterior branch of the superficial temporal artery is the most susceptible to injury and has an internal diameter of less than 1 mm [9]. The various layers that make up the temporal region can all be targeted for filler injection. One such method is to inject filler deep into the periosteal layer between the temporal bone and the temporal muscle [10]. This method works on the layer directly beneath the temporalis muscle and requires a large amount of filler at a deeper level than the muscle. However, this method may cause the filler to disperse within the muscle [11].

An alternative method, injection between the deep and superficial temporal fascia, has been proposed [2]. In the temporal region, the recommended injection point for the injection technique is 1 cm superior and 1 cm lateral to the lateral end of the eyebrow [12]. Compared to other points in the temporal region, this point is thought to be relatively safe in terms of blood vessels [13]. To ensure that there are no blood vessels during injection, the use of ultrasound guidance can improve the safety of this procedure [14].

Given the sensitivity and importance of filler injection in the temporal region, and the reduction of vascular complications, it is necessary that anatomical information such as vascular distribution, routes, and diameters is clearly defined. Therefore, the present study was conducted with the aim of determining the effectiveness of filler injection between the superficial and deep temporal fascia and the diameter of superficial temporal arteries for temple enlargement.

## 2 Method

### 2.1 Search Strategy

According to the PRISMA 2020 checklist [15], the current study was conducted by searching PubMed, EMBASE, and Web of Science databases up to July 2025 using relevant keywords; additional articles were found by reviewing the references and articles relevant to this review. Only English‐language articles were included. Titles, abstracts, and full texts of studies were assessed through separate literature searches by two independent, blinded authors.

The following Mesh terms were used to retrieve literatures:

(((((“Temporal Muscle”[Mesh] OR “Temporal Bone”[Mesh] OR “Temporal Lobe”[Mesh] OR “Mandibular Nerve”[Mesh] OR “Cranial Fossa, Middle”[Mesh]) AND “Dermal Fillers”[Mesh]) OR “Hyaluronic Acid”[Mesh]) AND (“Safety”[Mesh] OR “Safety Management”[Mesh])) AND “Temporal Arteries”[Mesh]) AND “Cannula”[Mesh].

### 2.2 Selection Criteria

#### 2.2.1 Inclusion Criteria

Studies that met the requirements of the PICO strategy were included:

Population (P): restoring volume to the sunken temple area with filler injections.

Intervention (I): Ha filler.

Comparison (C): not defined or no treatment group.

Outcome (O): treatment outcome.

#### 2.2.2 Exclusion Criteria

1. Reviews, in vitro studies, Animal studies, literature, Letters to the Editor, Congress Abstracts.
2. Studies with incomplete or scattered data, failure to report complete data in the text of the article, lack of access to data.
3. Published language of the article other than English.
4. Filler injections in areas other than the temples.
5. Failure to provide safety and effectiveness.

### 2.3 Data Extraction

Two researchers independently and blindly extracted data from each included study using a researcher‐made “Demographic and Primary Data Extraction” form; a third researcher resolved any disagreements between researchers. The data extraction form included the name of the first author, year of publication, sample size, methodology, and results.

### 2.4 Quality Assessment

Two authors independently assessed the quality of the studies. The Newcastle‐Ottawa Scale (NOS) was used to assess the quality of the included studies for cohort studies [16], and Version 2 of the Cochrane risk‐of‐bias tool for randomized trials (RoB 2) for RCT studies [17]. Three criteria (selection, comparability, and outcomes) were considered in the NOS tool and three criteria (trial design, conduct, and reporting) were considered in the RoB 2 tool. The NOS tool is scored from zero to nine (0–3, 4–6, and 7–9) and the RoB 2 tool is scored from zero to six (0–2, 3–4, 5–6). Scores of 7–9 for NOS and 5–6 for RoB 2 indicate low risk of bias.

### 2.5 Statistical Analysis

Statistical heterogeneity among studies was evaluated with the use of *I* ^2^ statistic and *Q* test *p*‐value < 0.05: No heterogeneity: 0.0% < *I* ^2^ < 24.9%; low heterogeneity: 25.0% < *I* ^2^ < 49.9%; moderate heterogeneity: 50.0% < *I* ^2^ < 74.9%; high heterogeneity: 75.0% < *I* ^2^ < 100%. STATA/MP.v17 (College Station, Texas, USA) was used to perform the analyses. The random effects model with restricted maximum–likelihood (REML) method was used to determine the efficacy.

The *I* ^2^ statistic and *Q* test *p*‐value < 0.05 were used to assess statistical heterogeneity among studies:

No heterogeneity: 0%–24.9%.

Low heterogeneity: 25%–49.9%.

Moderate heterogeneity: 50%–74.9%.

High heterogeneity: 75.0%–100%.

STAT/MP. Analysis was done using v17 (College Station, Texas, USA). The effectiveness of the random effect model was assessed using the Restricted Maximum Likelihood (REML) method.

## 3 Result

### 3.1 Literature Search

**FIGURE 1:**
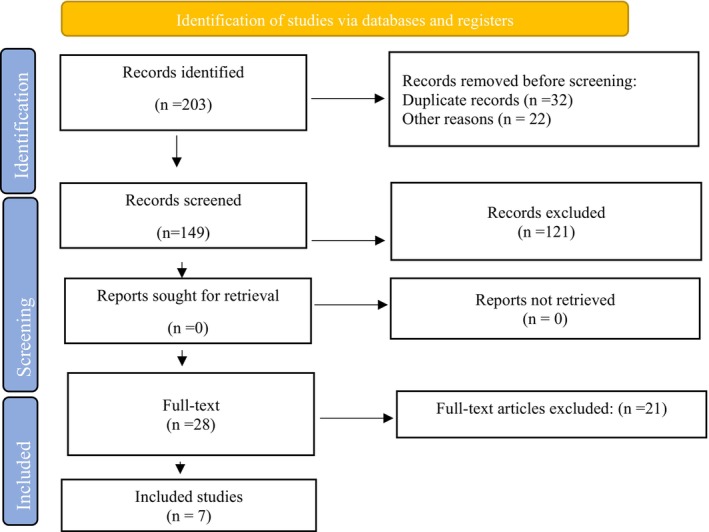
PRISMA 2020 Flow Diagram.

According to PRISMA 2020 Flow Diagram, after a comprehensive search based on keywords in international databases, 203 articles were found. After reviewing the titles of the articles, articles that did not meet the inclusion and exclusion criteria were excluded; the abstracts of 149 articles were reviewed and studies that did not meet the study selection criteria were excluded at this stage (*n* = 121); the full text of 28 articles was carefully reviewed by two blinded and independent authors, and disagreements were resolved by a third researcher; finally, after removing irrelevant articles, seven articles were included in the study for meta‐analysis (Figure 1).

### 3.2 Study Characteristics

Seven studies assessment the temple augmentation (a total sample size = 109). Table 1 shows a summary of the study data.

**TABLE 1:** Data extracted from included articles.

Study years	Sample size	Mean age (years)	Statistical population	Cannula type	HA filler volume (one side)	Injection site	Assessing the injection site and cannula position
Hong et al., 2025 [18]	1	NR	Female patient	21‐G	1 mL	STF and DTF	Ultrasound imaging system
Bae et al., 2024 [11]	1	30	Female patient	18‐G	1.5 mL	STF and DTF	Ultrasound imaging system
Kim et al. 2024 [4]	25	NR	Female patient	23‐G	1 mL	STF and DTF	Three‐dimensional scanning, hollowness examination, and sonographic measurements
Lee et al., 2022 [2]	50	38.22	Patients	21‐G	1.08 mL	STF and DTF	Doppler ultrasonography
Desyatnikova et al., 2022 [19]	1	48	Female patient	21‐G	1 mL	STF and DTF	Ultrasound image
Müller et al., 2021 [20]	30	53.1	Female patient	21‐G	2 mL	STF and DTF	3‐Dimensional surface imaging
Cotofana et al., 2021 [21]	1	NR	Female patient	21‐G	1 mL	STF and DTF	Ultrasound imaging

Abbreviations: DTF, deep temporal fascia; NR, not reported; STF, superficial temporal fascia.

### 3.3 Effectiveness

**FIGURE 2:**
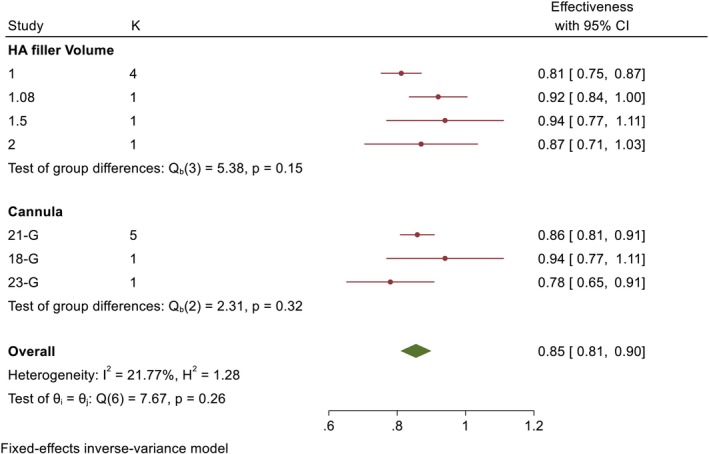
Forest plot showed subgroup meta‐analysis of the efficacy of HA filler injection in the STF and DTF.

The efficacy of HA filler injection in the STF and DTF area showed an efficacy of 85% (ES: 85%, 95% CI: 81%–91%) (Figure 2).

According to subgroup meta‐analysis, higher efficacy was observed with volume injection of 1.5 mL on each side (ES: 94%, 95% CI: 77%–100%); however, only one study had investigated this, and four studies investigating the efficacy of HA filler injection of 1 mL showed an efficacy of 81% (ES: 81%, 95% CI: 75%–87%) (Figure 2).

Subgroup meta‐analysis showed that the 18‐gauge cannula had the highest effectiveness (ES: 94%, 95% CI: 77%–100%); however, it was investigated in one study, and the 21‐gauge cannula, which was used in four studies, showed an effectiveness of 86% (ES: 86%, 95% CI: 81%–91%). These findings indicate that since the 18‐gauge cannula is 1.27 mm and the 21‐gauge cannula is 1.5 mm, the 18‐gauge cannula and 21‐gauge cannula can be considered a relatively safe approach for highlighting the temporal region (Figure 2).

According to the test of group differences, no statistically significant difference was observed between the injection volume (*p* = 0.15) and cannula (*p* = 0.32) on the effectiveness of injection in the temporal region (STF and DTF); however, using a smaller cannula can increase the safety of injection (Figure 2).

The *I* ^2^ = 21.77% coefficient indicates low heterogeneity between studies (*p* = 0.26) (Figure 2).

## 4 Discussion

The use of filler injections to improve the appearance of the temples has become increasingly popular over the past few decades due to its simple, minimally invasive procedure and long‐lasting results. However, the vascular complications and anatomical complexities of the temporal region should not be overlooked [22]. Evaluation of anatomical factors is of great importance in determining the efficacy and safety of filler injections. Studies have emphasized the anatomy of the superficial temporal artery and its vulnerability to procedures due to its reduced internal diameter, which is often less than 1 mm, especially on the temporal side [23].

The present meta‐analysis showed that an injected HA filler volume of 1 to 1.5 mL administered to each side was highly effective, and the use of 18‐gauge and 21‐gauge cannulas, due to their thinner size, also has high efficacy with higher safety. The difference in cannula size and arterial diameter indicates a relatively safe method for augmenting the temporal region.

In clinical settings, temporal augmentation is performed by injecting fillers into different layers of the temporal region. There are methods that target the periosteal layer between the superficial and deep layers of the temporal fascia or between the temporal bone and the temporal muscle. Injecting the deep periosteal layer increases volume, but also carries the risk of the filler spreading throughout the muscle, which can lead to nodules. When injected between the superficial and deep layers of the temporal fascia, a smaller amount of filler can be used for augmentation, which is thought to be safer and more effective. When injecting temporal filler, doctors typically target a precise injection site that is above the lateral end of the eyebrow and approximately 1 cm laterally. This reference point, while relatively safer, may vary from person to person, highlighting the importance of customized procedures. To further improve the safety of the procedure, ultrasound guidance is used to confirm the absence of blood vessels during injection [11]. However, even with ultrasound guidance, there is a potential risk of injuring the deep temporal artery in this area, and caution should be exercised.

## 5 Conclusion

For temple augmentation, hyaluronic acid filler injection between the superficial and deep temporal fasciae is a highly effective and safe procedure; it is also recommended to inject filler in small volumes and to use 18‐gauge and 21‐gauge cannulas to target the supraperiosteal and supra‐deep temporal fascia layers; ultrasound imaging is also important.

## Funding

The authors have nothing to report.

## Ethics Statement

The authors have nothing to report.

## Consent

The authors have nothing to report.

## Conflicts of Interest

The authors declare no conflicts of interest.

## Data Availability

The data that support the findings of this study are available on request from the corresponding author. The data are not publicly available due to privacy or ethical restrictions.
